# Sunitinib Exerts *In Vitro* Immunomodulatory Activity on Sarcomas *via* Dendritic Cells and Synergizes With PD-1 Blockade

**DOI:** 10.3389/fimmu.2021.577766

**Published:** 2021-02-26

**Authors:** Darina Ocadlikova, Mariangela Lecciso, Javier Martin Broto, Katia Scotlandi, Michele Cavo, Antonio Curti, Emanuela Palmerini

**Affiliations:** ^1^ IRCCS Azienda Ospedaliero-Universitaria di Bologna, Istituto di Ematologia “Seràgnoli”, Bologna, Italy; ^2^ Dipartimento di Medicina Specialistica, Diagnostica e Sperimentale, Università di Bologna, Bologna, Italy; ^3^ Virgen del Rocio University Hospital, Institute of Biomedicine Research (IBIS), Seville, Spain; ^4^ Laboratory of Experimental Oncology, IRCCS Istituto Ortopedico Rizzoli, Bologna, Italy; ^5^ Department of Experimental, Diagnostic and Specialty Medicine (DIMES), Osteoncology, Bone and Soft Tissue Sarcomas and Innovative Therapies, IRCCS Istituto Ortopedico Rizzoli, Bologna, Italy

**Keywords:** osteosarcoma, synovial sarcoma, tyrosine kinase inhibitor (TKI), nivolumab (PubChem SID: 178103907), sunitinib (PubChem CID: 5329102), immunomodulation, dendritic cell (DC), T regulatory cells (Tregs)

## Abstract

**Background:**

High-grade sarcomas are a heterogeneous group of aggressive tumors arising in bone and soft tissues. After relapse, treatment options are limited. The multi-targeted receptor tyrosine kinase inhibitors (TKIs) sunitinib and inhibitor of PD-1 (anti-PD-1) nivolumab have shown antitumor activity in selected subtypes. In this study, we examine the role of TKIs and PD-1 based therapy in *in vitro* cocultures of sarcoma.

**Methods:**

The human osteosarcoma (SaOS-2) and synovial sarcoma (SYO-1) cell lines were treated with sunitinib. After cell death and proliferation assessment, expression of PD-L1 was analyzed by flow cytometry. Sunitinib-treated sarcoma cells were cocultured with dendritic cells (DCs), and the phenotype of mature DCs was determined by flow cytometry. Mature DCs were cultured with autologous T cells. PD-1 expression on T cells, their proliferation, T regulatory cell (Tregs) induction and IFN-γ production, before and after nivolumab exposure, were analyzed.

**Results:**

Along with its anti-proliferative and direct pro-apoptotic effect on sarcoma cell lines, sunitinib prompted PD-L1 upregulation on sarcoma cells. Interestingly, sunitinib-treated sarcoma cells drive DCs to full maturation and increase their capacity to induce sarcoma-reactive T cells to produce IFN-γ. Conversely, no effect on T cell proliferation and T cell subpopulation composition was observed. Moreover, both bone and synovial sarcoma cell lines induced Tregs through DCs but sunitinib treatment completely abrogated Treg induction. Finally, sarcoma cell lines induced PD-1 upregulation on both effector T cells and Tregs when loaded into DCs, providing a rationale for using PD-1 blockade. Indeed, PD-1 blockade by nivolumab synergized with sunitinib in inducing IFN-γ-producing effector T cells.

**Conclusions:**

Taken together, our *in vitro* data indicate that the treatment of sarcoma cells with sunitinib can exert significant changes on immune cell subsets toward immune activation, leading to DC-based cross-priming of IFN-γ-producing effector T cells and reduced Treg induction. PD-1 blockade with nivolumab has a synergistic effect with sunitinib, supporting the use of TKI and anti-PD-1 approach in sarcomas, and perhaps in other cancers. DC-targeted drugs, including toll-like receptor 3 inhibitors and CD47 inhibitors, are under development and our preclinical model might help to better design their clinical application.

## Background

High grade sarcomas are a rare, aggressive and heterogeneous group of mesenchymal neoplasms of bone and soft tissue. Whereas there is agreement that surgery, with adjuvant chemotherapy in the majority of cases, is paramount for the outcome, 30–50% of patients without evident metastases at presentation will die of the disease (1, 2). There is thus an urgent need to identify novel treatment methods.

Immunotherapy is part of the therapeutic armamentarium in several solid tumors. PD-L1 expression is low in sarcomas, associated with poor prognosis (3–5) and predictive of response to checkpoint inhibitors (6, 7). The activity of checkpoint inhibitor monotherapy (6) was limited overall, with better activity in selected histotypes such as undifferentiated pleomorphic sarcomas or dedifferentiated liposarcomas (6), alveolar soft part sarcomas (8) and dedifferentiated chondrosarcomas (6).

Sunitinib is a small molecule receptor tyrosine kinase (RTK) inhibitor that blocks signaling of multiple RTKs, including vascular endothelial growth factor (VEGF) and platelet-derived growth factor (PDGF) receptors (9), with pre-clinical evidence of activity in metastasizing osteosarcomas (10). Sunitinib is approved by the Food and Drug Administration for the treatment of patients with gastrointestinal stromal tumors (11) and has been examined in combination with nivolumab in selected soft tissue sarcomas such as alveolar sarcomas or dedifferentiated bone chondrosarcomas (8, 12). Sunitinib may exert immunostimulatory activity through the modulation of a ratio of immunostimulatory versus immunoregulatory cells. In fact, in addition to its anti-angiogenic function, sunitinib has immune modulating properties, which include increasing the influx of lymphocytes and DCs into the tumor, while decreasing intratumoral frequencies of Tregs and myeloid-derived suppressor cells (MDSCs) (13–15). On the other hand, in renal cell carcinoma (RCC) studies, sunitinib increases PD-L1 expression in tumor cells and Treg infiltration (16). In this study, we hypothesized that PD-1/PD-L1 immune checkpoint inhibitors may reduce cancer immunological tolerance and that immunomodulation with a TKI might improve this activity. Therefore, we characterized bone and soft tissue sarcoma cell lines after treatment with sunitinib and nivolumab. To assess the effect of this combination, we generated DCs pulsed with sarcoma cells and describe T cell and DC functional changes after treatment with (1) sunitinib, (2) nivolumab and (3) sunitinib with nivolumab, including T-cell subpopulation composition, T-cell proliferation and IFN-γ production.

## Materials and Methods

### Human Cells

CD3^+^ and CD14^+^ cells were purified by magnetic separation (MiltenyiBiotec, Bergisch Gladbach, Germany), according to the manufacturer’s instructions, from mononuclear cells separated from buffy coats of healthy donors by Ficoll-Hypaque centrifugation (Amersham, Buckinghamshire, United Kingdom) after informed consent signature (local Ethics Committee approval code 94/2016/O/TES). Purity of cell populations was always > 90%.

### Cell Lines

Human osteosarcoma cell lines SaOS-2 were obtained from Dr. Massimo Serra’s Laboratory (IRCCS Istituto Ortopedico Rizzoli, Bologna, Italy) and cultured in Dulbecco’s Modified Eagle Media supplemented with 10% heat-inactivated fetal bovine serum (FBS; Sigma Aldrich, St. Louis, USA), 2 mM L-glutamine, 100 U/ml penicillin, and 100 µg/ml streptomycin (all from MP Biomedicals, Milan, Italy) at 37°C humidified atmosphere with 5% CO_2_. Human synovial sarcoma cell line SYO-1, bearing the pathogenetic translocation (X;18)(p11.2;q11.2), was obtained from Dr Akira Kawai (National Cancer Center, Tokyo, Japan) and Dr. Aki Yoshida Laboratories (Okayama University, Tokyo, Japan) and cultured in Iscove’s Modified Dulbecco’s Media supplemented with 10% heat-inactivated FBS (Sigma Aldrich), 2 mM L-glutamine, 100 U/ml penicillin, and 100 µg/ml streptomycin (all from MP Biomedicals) at 37°C humidified atmosphere with 5% CO_2_. The cells were split twice a week. Before cell treatment or usage, SaOS-2 and SYO-1 were removed from culture flasks using 0.05% trypsin-ethylenediamine tetraacetic acid (Life Technologies, Waltham, USA) and washed in PBS. After cell-counting, the cells were resuspended in PBS or cell culture medium.

### Drugs

Sunitinib/Sutent^®^/PF-00262192 is an oral multitargeted TKI (including VEGFR, kit and PDGFR) and was provided by Pfizer Inc, NJ, USA. Nivolumab/Opdivo^®^ is a humanized, immunoglobulin G4 mAb to PD-1 and was provided by Bristol Myers Squibb (NYC, NY, USA).

### Apoptosis of Sarcoma Cell Lines

SaOS-2 and SYO-1 cell lines in exponential phase of growth were harvested, seeded (150,000 cells/ml; 24-well plate) and incubated for at least 1 hour at 37°C humidified atmosphere with 5% CO_2_ to guarantee adherence. The cells were then treated for 24 h with sunitinib at various concentrations (10, 15, 2,0 or 30 μM) to test apoptosis using Annexin-V-FLUOS Apoptosis Detection Kit (Roche, Basel, Switzerland), according to the manufacturer’s instructions. The percentage of apoptotic cells was determined using an Accuri C6 flow cytometer (BD Biosciences, Franklin Lakes, USA) and at least 10,000 events were analyzed by FCS Express 4 Research Edition software. Untreated cells with dimethyl sulfoxide (DMSO) were used as negative control and unstained cells were used as fluorescence control.

### Proliferation of Sarcoma Cell Lines

SaOS-2 and SYO-1 cell lines in exponential phase of growth were harvested, seeded (5,000 cells/100 μl; 96-well plate) and incubated for 1 hour at 37°C humidified atmosphere with 5% CO_2_ to guarantee adherence. The cells were then treated for 24, 48, or 72 h with sunitinib at various concentrations (0 + DMSO, 0.5, 1, 3, 5, 7, or 10 μM) to test proliferation using a CellTiter 96^®^ Aqueous One Solution Cell Proliferation Assay (MTS) (Promega, Madison, USA). After 24, 48 or 72 h, the cells were treated with 20μl of CellTiter 96^®^ Aqueous One Solution Reagent. After 3 h of incubation in the dark, the absorbance at 490 nm was detected by a 96-well plate reader (Multiskan EX Thermo Fisher Scientific, Waltham, USA). For each experiment, a standard curve was measured in order to calculate the cell number of each well plate.

### PD-L1 Expression on Sarcoma Cell Lines

Untreated or sunitinib-treated SaOS-2 and SYO-1 cell lines (20 and 15 μM sunitinib for 24 h, respectively) were harvested and stained for PD-L1 expression by flow cytometry. Briefly, 100,000 cells were stained with anti-human CD274/PD-L1 APC (clone B7-H1; eBiosciences/ThermoFisher, San Diego, USA) mAb and incubated at 4°C for 20 min in the dark, then washed and resuspended in PBS. The percentage of PD-L1 positive cells was determined using the FACS Canto II flow cytometer (BD Biosciences) and at least 10,000 events were analyzed by FCS Express 4 Research Edition software. Untreated cells were used as negative control and unstained cells were used as fluorescence control.

### Immunophenotype of DCs

Human monocyte-derived immature DCs were generated from a 6-day culture of CD14^+^ cells in RPMI 1640 medium (Lonza, Milan, Italy), supplemented with 10% heat-inactivated FBS (Sigma Aldrich), 2 mM L-glutamine, 100 U/ml penicillin, and 100 µg/ml streptomycin (all from MP Biomedicals) (complete RPMI), and maintained at 37°C humidified atmosphere with 5% CO_2_ in the presence of 50 ng/ml of granulocyte-macrophage colony-stimulation factor (GM-CSF; Endogen, Way Woburn, USA) and 800 U/ml of IL-4 (MiltenyiBiotec), as previously described (17, 18). After 6 days, immature DCs were harvested and used for DC loading. In particular, the DCs were mixed or not with untreated (irradiated at 3,000 cGy) or sunitinib-treated SaOS-2 (20 μM) or SYO-1 (15 μM) cells for 24 h at a ratio of 2:1 (SaOS-2/SYO-1:DC) in RPMI complete medium at a concentration of 0.5 × 10^6^ DCs/ml. After 24 h, loaded and unloaded DCs were stained for 15 min in the dark using the following anti-human mAbs: CD1a PE-Cy7 (clone HI149; Biolegend, San Diego, USA) CD86 PE-Cy7 (clone IT2.2; eBioscience/ThermoFisher), CD80 APC (clone 2D10; Biolegend), CD83 PE (clone HB15; Biolegend), CD197/CCR7 Alexa Fluor 647 (clone G043H7; Biolegend), CD274/PD-L1 APC (clone B7-H1; eBiosciences/ThermoFisher) and CD40 PerCP/Cy5.5 (clone HB14; Biolegend). For each sample, unstained DCs were used as negative fluorescence control. At least 10,000 events of each sample were collected and analyzed for immunophenotype at FACS Canto II Flow Cytometer (BD Biosciences). DCs were then used as a stimulus for T cells to test T-cell proliferation (2.8.), subpopulation composition (2.9.), IFN-γ production (2.10.) and Treg induction (2.11.).

### T-Cell Proliferation

One thousand DCs were prepared as described in 2.7 and irradiated at 30 Gy. DCs were then cocultured with 150,000 carboxyfluorescein succinimidyl ester (CFSE)-labeled autologous CD3^+^ T cells. Briefly, autologous CD3^+^ T cells were labeled by CFSE (CellTrace™ CFSE Cell Proliferation Kit, Thermofisher) at a concentration of 5 µM for 20 min at 37°C humidified atmosphere with 5% CO_2_. After incubation, the cells were washed with PBS and seeded at a concentration of 1 × 10^6^ T cells/ml in flat-bottom 96-well microplates (150,000 cells/well) and irradiated DCs were added. After 5 days of coculture, T cells were harvested, washed with PBS and T-cell proliferation by flow cytometry was evaluated. As positive control, CD3^+^ T cells stimulated for 3 days with phytohemagglutinin (PHA; Sigma Aldrich) at a concentration of 20 g/ml were used. Unstimulated CD3^+^ T cells were used as negative control and unstained CD3^+^ T cells were used as negative fluorescence control. At least 10,000 events of each sample were collected and analyzed at FACS Canto II Flow Cytometer (BD Biosciences). Proliferation index was calculated using FCS Express 4 Research Edition software.

### T-Cell Subpopulations

Fifteen thousand DCs were prepared as described in 2.7 and cocultured with 150,000 autologous CD3^+^ T cells at a concentration of 1 × 10^6^ T cells/ml. After 5 days of coculture, T cells were harvested, washed with PBS and stained for 15 min at the dark using the following anti-human mAbs: CD3 APC-H7 (clone SK7, BD Biosciences), CD4 Pe-Cy7 (clone SK-3, Thermofisher), CD8 PE (clone SK1, BD Biosciences), CD279/PD-1 APC (clone MIH4, Thermofisher), CD197/CCR7 Alexa Fluor 647 (clone G043H7; Biolegend), and CD45RA V500 (clone HI100; Biolegend). Unstimulated CD3^+^ T cells were used as negative control, and unstained CD3^+^ T cells were used as negative fluorescence control. At least 10,000 events of each sample were collected and analyzed at FACS Canto II Flow Cytometer (BD Biosciences).

### IFN-γ Production by T Cells

Effector CD3^+^ T cells were cocultured for 7 days in complete RPMI enriched with 10 U/ml of IL-2 (Roche) at a concentration of 1 × 10^6^ T cells/ml, with (i) no stimulus, (ii) autologous DCs loaded with untreated SaOS-2/SYO-1 cells irradiated at 30 Gy at a ratio of 1:10 (DC:T cell) or (iii) autologous DCs loaded with SaOS-2/SYO-1 cells treated with sunitinib (as described in 2.7) at a ratio of 1:10 (DC:T cell), then restimulated in the same manner, and 24 h after the restimulation, the IFN-γ production was tested. Briefly, each type of T effector cells was cultured at a concentration of 1 × 10^6^ T cells/ml alone as negative effector control, with Ionomycin (IM; 500 ng/ml; Sigma-Aldrich) and Phorbol-12-Myristate-13-acetate (PMA; 10 ng/ml; Sigma-Aldrich) as positive effector control in the presence or absence of nivolumab (20 µg/ml), or with the following 3 types of targets (10:1 ratio) for 4 h: (i) unloaded autologous DCs (as negative target control), DCs loaded with diverse sarcoma cell lysate (obtained after three cycles of cell freeze-thawing and filtering through an insulin syringe) with respect to effector stimulation (as *nonspecific* target control), or DCs loaded with the same sarcoma cell lysate (obtained after three cycles of cell freeze-thawing and filtering through an insulin syringe) with respect to effector stimulation (as a *specific* target). Brefeldin A (2 μg/ml; BD Biosciences) was then added in each well to stop and fix the IFN-γ production. After 12 h of incubation at 37°C humidified atmosphere with 5% CO_2_, the effector T cells were stained for 15 min in the dark with the following surface anti-human mAbs: CD4 FITC (clone RPA-T4; Thermofisher) and CD8 APC (clone SK1). T cells were then washed with PBS and fixed with 4% paraformaldehyde (Sigma-Aldrich) for 10 min at room temperature. After washing twice with 0.1% saponin (Sigma-Aldrich) to permeabilize them, intracellular staining with anti-human IFN-γ PE (clone 4S.B3; Thermofisher) was performed for 30 min at 4°C. After washing twice with 0.1% saponin (Sigma-Aldrich), the cells were analyzed by flow cytometry. Unstained CD3^+^ T cells were used as negative fluorescence control. At least 10,000 events of each sample were collected and analyzed at FACS Canto II Flow Cytometer (BD Biosciences).

Twenty thousand DCs were prepared as described in 2.7 and cocultured with 200,000 autologous CD3^+^ T cells at a concentration of 1 × 10^6^ T cells/ml. After 5 days of coculture, T cells were harvested, washed with PBS and stained for 15 min in the dark using the following anti-human mAbs: CD4 APCH7 (clone SK3; BD Biosciences), CD25 PeCy7 (clone BC96; Biolegend), CD127 PerCP 5.5 (clone A019D5; Biolegend) and PD-1 APC (clone EH12.2H7; Biolegend). Intracellular staining of FOXP3 using Foxp3/Transcription Factor Staining Buffer Set (eBioscience/Thermofisher) was performed as follows. Unstimulated CD3^+^ T cells were used as negative control and unstained CD3^+^ T cells were used as negative fluorescence control. At least 5,000 events of Tregs in each sample were collected and analyzed at FACS Canto II Flow Cytometer (BD Biosciences).

### Statistical Analysis

Data are expressed as mean ± standard error of mean (SEM) of values obtained in the experiments. Statistical analyses were performed with GraphPad Prism 6 software (GraphPad Software, Inc., La Jolla, USA), using ANOVA or unpaired t-test. P values < 0.05 were considered statistically significant.

## Results

### Sunitinib Inhibits the Proliferation of Sarcoma Cells by Increasing Apoptosis and Concomitantly Upregulates Their Basal Expression of PD-L1

**Figure 1:**
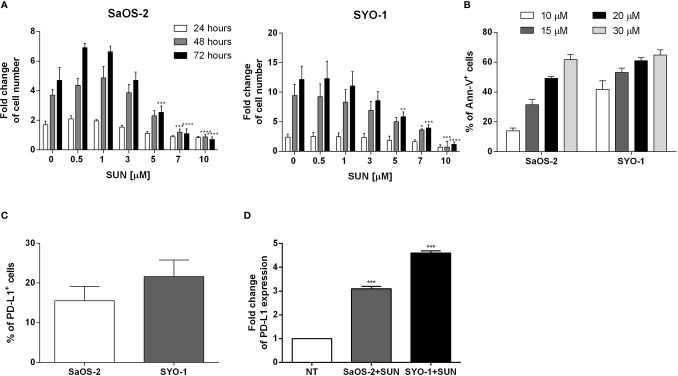
Analysis of sarcoma cell proliferation, apoptosis and PD-L1 expression before and after sunitinib treatment. **(A)** SaOS-2 and SYO-1 cell lines were treated for 24, 48, or 72 h with sunitinib (SUN) at various concentrations (0, 0.5, 1, 3, 5, 7 and 10 μM). The cell number was determined by colorimetric assay. Untreated cells (DMSO + 0 μM sunitinib) were used as negative control. The values are represented as mean ± SEM of 5 independent experiments. *p < 0.05; **p < 0.01; ***p < 0.001; ****p < 0.0001 compared to untreated cells. **(B)** SaOS-2 and SYO-1 cell lines were treated for 24 h with sunitinib at various concentrations (10, 15, 20 and 30 μM) and analyzed by flow cytometry. The percentages of apoptotic Ann-V^+^ cells were calculated as differences between treated and untreated cells + DMSO. The concentrations which made it possible to achieve an apoptosis level of approximately 50% were chosen for the following functional experiments (20 or 15 μM sunitinib for SaOS-2 or SYO-1, respectively). The values are represented as mean ± SEM of 5 independent experiments. **(C)** Flow cytometry analysis of PD-L1 expression before and **(D)** after 24 h of sunitinib treatment at concentrations of 20 μM for SaOS-2 and 15 μM for SYO-1 cell lines (SaOS-2+SUN and SYO-1+SUN). The percentage of PD-L1 expression was expressed as fold change. Untreated cells (no treatment; NT) were used as reference and set at 1. The values are represented as mean ± SEM of 5 independent experiments. *p < 0.05; **p < 0.01; ***p < 0.001; ****p < 0.0001 compared to untreated cells.

The effect of sunitinib on osteosarcoma and synovial sarcoma cells was characterized. Firstly, we tested its effect on proliferation of SYO-1 and SaOS-2 cell lines. The treatment of SaOS-2 and SYO-1 cell lines for 24, 48, and 72 h with 0.5, 1, 3, 5, 7, and 10 μM sunitinib inhibited cell proliferation in a dose-dependent manner, as assessed by cell proliferation-assay (**Figure 1A**), with optimal results obtained after 48 h with 7 μM sunitinib in SaOS-2 (p < 0.001) and 10 μM in SYO-1 (p < 0.001) cell lines.

Accordingly, the treatment of SaOS-2 and SYO-1 cell lines for 24 h with 10, 15, 20 and 30 μM sunitinib increased apoptosis in a dose-dependent manner, as assessed by cell apoptosis-assay (**Figure 1B**), with optimal results obtained with 20 μM sunitinib in SaOS-2 (49.2 ± 1.2% of AnnV^+^ cells) and 15 μM sunitinib in SYO-1 (53.2 ± 2.8%) cell lines after 24 h. Given the immunomodulatory effect of TKIs, including sunitinib, on PD-L1 expression in RCC cells (16, 19), we tested the effect of sunitinib on PD-L1 expression on sarcoma cells. At baseline, the PD-L1 expression was detected in both untreated sarcoma cell lines, with a mean expression of 15.5 ± 3.6% for SaOS-2 and 21.6 ± 4.2% for SYO-1 cell lines (**Figure 1C**). As shown in **Figure 1D**, after 24 h of sunitinib treatment (20 and 15 μM for SaOS-2 and SYO-1, respectively), the PD-L1 expression significantly increased in both sarcoma cell lines (fold change 3.1 ± 0.1% for SaOS-2 and 4.6 ± 0.1% for SYO-1, respectively) compared to untreated cells (p < 0.001 for both cell lines). These findings confirmed previous reports indicating that both bone SaOS-2 and synovial SYO-1 sarcoma cell lines are sensitive to sunitinib treatment in terms of increased apoptosis and decreased proliferation. Interestingly, our data on the capacity of sunitinib to increase PD-L1 expression on sarcoma cells revealed a potential role of sunitinib as an immunomodulatory agent and prompted us to further characterize *in vitro* its effect on different immune cell subsets.

### After Sunitinib Treatment, Dying Sarcoma Cells Are Potent Inducers of Full DC Maturation

After treatment with antineoplastic agents, the cross-priming of antigen-specific T cells *via* DCs represents a crucial effect on the induction of an effective anti-tumor immune response (Zhou 2019 and Ocadlikova 2019). We next sought to investigate the capacity of sunitinib to affect DC phenotype and function after sarcoma cell line killing. SaOS-2 and SYO-1 cell lines were treated for 24 h with 20 and 15 μM sunitinib, respectively, and used for DC pulsing. After 24 h, DC maturation status was evaluated by testing the surface expression of CD1a, CD80, CD83, CD86, CD40, CCR7 and their correlation with PD-L1, which was shown to be upregulated after sunitinib treatment.

**Figure 2:**
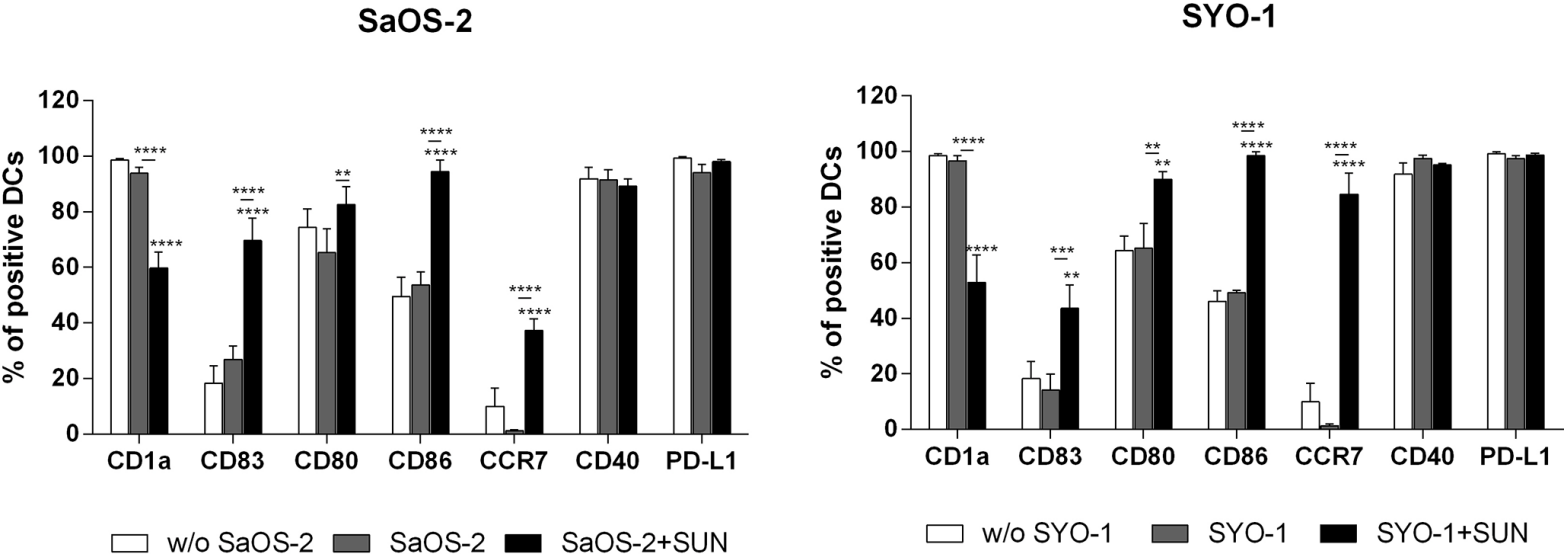
Flow cytometry analysis of DC-maturation. SaOS-2 and SYO-1 cell lines were treated (SaOS-2+SUN and SYO-1+SUN) or left untreated (SaOS-2 and SYO-1) for 24 h with sunitinib (SUN) at concentrations of 20 μM for SaOS-2 and 15 μM for SYO-1 cell lines, and used for monocyte-derived DC pulsing for another 24 h at a ratio of 1:2 (DC:sarcoma cell). DCs pulsed with untreated sarcoma cells were irradiated. The immunophenotype was analyzed by flow cytometry. Unloaded DCs (w/o SaOS-2/SYO-1) were used as negative control. The values are represented as mean ± SEM of 5 independent experiments. **p < 0.01; ***p < 0.001; ****p < 0.0001 compared to unloaded DCs.

As shown in **Figure 2**, sunitinib treatment induced a significant upregulation of co-stimulation molecules CD80 and CD86 in both sarcoma cell lines (p < 0.01 and p < 0.0001, respectively) when compared to DCs loaded with untreated sarcoma cells. A similar pattern was observed for the upregulation of DC-maturation marker CD83 (69.6 ± 8.2% with p < 0.0001 and 43.6 ± 8.4% with p < 0.01 for SaOS-2 and SYO-1, respectively) and CCR7, that is required for DC migration to lymph nodes (37.3 ± 4.1%; p < 0.0001 and 84,6 ± 7.6%; p < 0.0001, respectively), compared to unloaded DCs. Both CD83 and CCR7 upregulation was significant also when compared to DCs loaded with untreated sarcoma cells. Moreover, a significant decrease was observed of CD1a, a molecule associated with lipid antigen processing and expression on immature DCs, when compared to both unloaded DCs and DCs loaded with untreated cells (p < 0.0001). Taken together, sunitinib treatment of both bone and synovial sarcoma cells induced a significant upregulation of CD80, CD86, CD83 and CCR7 which are essential for full DC maturation.

### Sunitinib Treatment of Synovial Sarcoma Cells Elicited DC-Based Cross-Priming Effect of IFN-γ-Producing Effector T Cells

To determine the effect of sunitinib treatment of sarcoma cells on T effector cells through DC pulsing, a set of further experiments was performed, including the analysis of T-cell subpopulation composition and T-cell proliferation and IFN-γ production.

**Figure 3:**
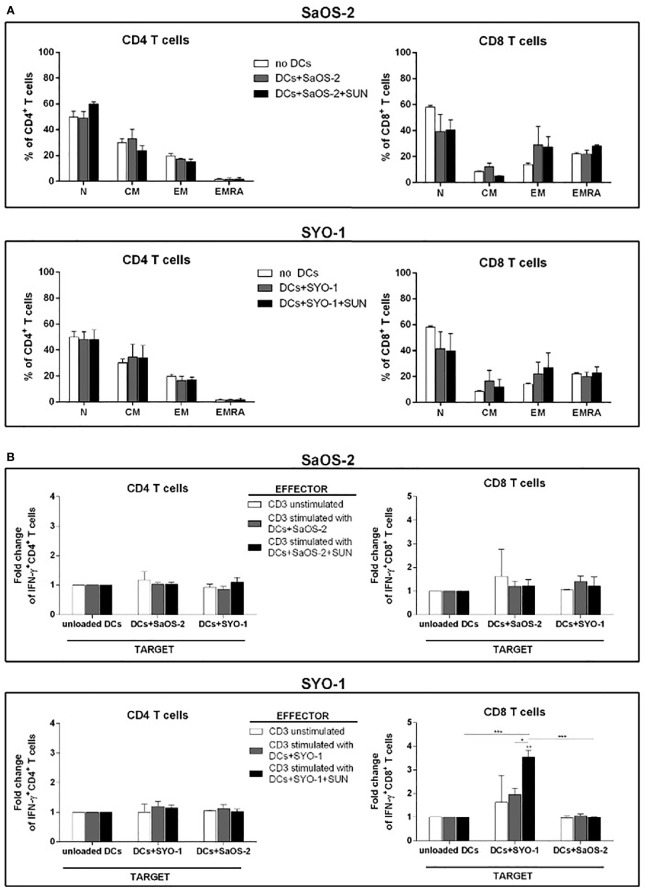
Flow cytometry analysis of T-cell subpopulations and IFN-γ producing T cells induced by DCs loaded with sunitinib-treated sarcoma cells. SaOS-2 and SYO-1 cell lines were left untreated or treated for 24 h with sunitinib (SUN) at concentrations of 20 μM and 15 μM, respectively, and used for monocyte-derived DC pulsing for another 24 h at a ratio of 1:2 (DC:sarcoma cell). DCs loaded with untreated and irradiated SaOS-2/SYO-1 (DCs+SaOS-2/SYO-1) or sunitinib-treated cells (DC+SaOS-2/SYO-1+SUN) were used to stimulate autologous CD3^+^ effector T cells at a ratio of 1:10 (DC:T cell). **(A)** After 5 days, the percentages of naïve (N), central memory (CM), effector memory (EM) and terminally differentiated EM expressing RA (EMRA) in both CD4^+^ and CD8^+^ T cells induced by DCs loaded with SaOS-2 or SYO-1 cell lines were evaluated by flow cytometry. Unstimulated CD3^+^ T cells (no DCs) were used as negative control. The values are represented as mean ± SEM of 5 independent experiments. **(B)** After 7 days, the T-cell stimulation was repeated (restimulation). Unstimulated CD3^+^ effector T cells were used as negative effector control. 24 h after restimulation, the IFN-y test was performed (see *IFN-γ Production by T Cells*) and the percentages of IFN-γ-producing CD3/CD4^+^ and CD3/CD8^+^ SaOS-2- or SYO-1-reactive effector T cells were evaluated by flow cytometry and expressed as fold change. DCs loaded with SaOS-2 and SYO-1 cell lysate (DC+SaOS-2 and DC+SYO-1; target) were used as *specific* (the same cell line with respect to effector stimulus) and *unspecific* (different cell line with respect to effector stimulus) targets, while unloaded DCs were used as negative target control and the values of IFN-γ-producing T cells against unloaded DCs were used as reference and set at 1. The values are represented as mean ± SEM of 5 independent experiments; *p < 0.05; **p < 0.01; ***p < 0.001.

Firstly, the composition of T-cell subpopulations was analyzed. For this purpose, sunitinib-treated SaOS-2 (20 μM) and SYO-1 (15 μM) sarcoma cell lines were pulsed into DCs, which in turn were used to stimulate T cells. After 5 days of coculture, Naïve (N), Central Memory (CM), Effector Memory (EM) and terminally differentiated EM expressing RA (EMRA) T cells were identified through CCR7 and CD45RA expression. In particular, CCR7^+^CD45RA^+^, CCR7^+^CD45RA^-^, CCR7^-^CD45RA^-^, and CCR7^-^CD45RA^+^ populations were analyzed as N, CM, EM, and EMRA T cells, respectively, by flow cytometry. As shown in **Figure 3A**, no changes in T-cell subpopulations were observed when DCs loaded with both untreated and sunitinib-treated SaOS-2 and SYO-1 cell lines were used for T-cell stimulation. The capacity to induce T-cell proliferation was then tested. DCs matured by sunitinib-treated SaOS-2 and SYO-1 cell lines were used for mixed lymphocyte culture with autologous T cells to determine their proliferative capacity. After 5 days of coculture, the proliferation index of T cells was determined. Only a weak proliferation of CD3^+^ T cells was observed when stimulated with DCs loaded with both untreated SaOS-2 and SYO-1 sarcoma cells (compared to unstimulated T cells), but no further proliferation was detected when sunitinib-treated SaOS-2 or SYO-1 cell lines were used (data not shown). These results could suggest some difficulties in T-cell stimulation mechanism through DCs after pulsing with sunitinib-treated sarcoma cells.

To further characterize the effect of sunitinib treatment of sarcoma cells on the functionality of pulsed DCs, sunitinib-treated SaOS-2 (20 μM) and SYO-1 (15 μM) were loaded into DCs, which were in turn used to stimulate and restimulate autologous CD3^+^ T cells for inducing sarcoma-specific T cells. As shown in **Figure 3B**, DCs loaded with sunitinib-treated SYO-1 cells were more efficient than DCs loaded with sunitinib-treated SaOS-2 cells in inducing sarcoma-reactive CD8^+^ T cells. In particular, CD8^+^ effector T cells stimulated by DCs loaded with sunitinib-treated SYO-1 cells and used against a *specific* target represented by DCs loaded with SYO-1 cell-lysate, achieved a significant 3.7 ± 0.3% fold change of IFN-γ producing CD8^+^ T cells when compared to unloaded DCs used as a target (fold change 1; p < 0,01) or DCs loaded with a *nonspecific* target represented by SaOS-2 cell-lysate (fold change 0.97 ± 0.03%; p < 0.01) (**Figure 3B**). Of note, neither SaOS-2 nor SYO-1 sunitinib-treated cells induced activation of CD4^+^ T cells by loading into DCs (**Figure 3B**).

Taken together, our *in vitro* data indicate that the treatment with sunitinib gives rise to increased immunogenicity of sarcoma cells, leading to elicited DC-based cross-priming effect of IFN-γ -producing effector T cells. Interestingly, synovial sarcoma cells were more effective than bone sarcoma cells in inducing IFN-γ production after sunitinib treatment.

### Sunitinib Prevents DC-Mediated Treg Induction by Sarcoma Cell Lines

**Figure 4:**
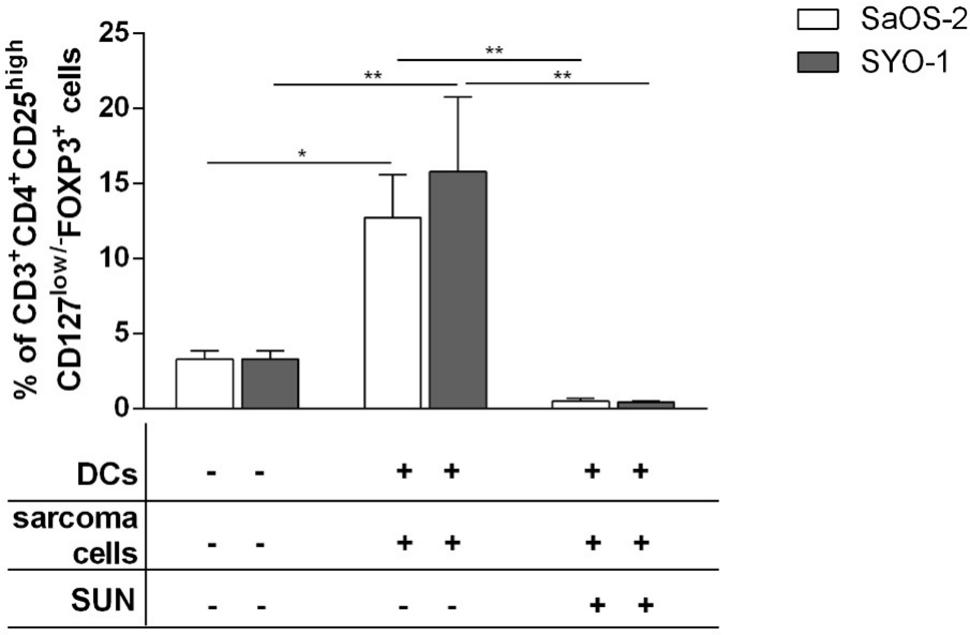
Flow cytometry analysis of Tregs induced by DCs loaded with sunitinib treated sarcoma cells. SaOS-2 and SYO-1 cell lines were left untreated or treated for 24 h with sunitinib (SUN) at concentrations of 20 μM and 15 μM, respectively, and used for monocyte-derived DC pulsing for another 24 h at a ratio of 1:2 (DC:sarcoma cell). DCs pulsed with untreated sarcoma cells were irradiated. DCs loaded with untreated SaOS-2/SYO-1 or sunitinib-treated cells were then mixed with autologous CD3^+^ T cells at a ratio of 1:10 (DC:T cell). Unstimulated CD3^+^ T cells were used as negative control. After 5 days, the percentage of CD3^+^CD4^+^CD25^high^CD127^low/-^FOXP3^+^ Tregs was evaluated by flow cytometry. The values are represented as mean ± SEM of 5 independent experiments. *p < 0.05; **p < 0.01.

The limited effect of DCs loaded with sunitinib-treated bone and synovial sarcoma cells on T-cell proliferation and subpopulation composition prompted us to also analyze Treg induction. SaOS-2 and SYO-1 treated with sunitinib (SaOS-2 with 20 μM and SYO-1 with 15 μM sunitinib, respectively) were pulsed into DCs which in turn were used to stimulate autologous CD3^+^ T cells. After 5 days, the induction of Tregs characterized as CD3^+^CD4^+^CD25^high^CD127^low/-^FOXP3^+^ T cells was evaluated. Both sarcoma cell lines loaded in DCs induced Tregs. In particular, DCs loaded with SaOS-2 or SYO-1 induced 12.7 ± 2.9% (p < 0.05) or 15.8 ± 5% (p < 0.01) of Tregs, respectively (**Figure 4**). Interestingly, as shown in **Figure 4**, Treg induction by both SaOS-2 or SYO-1 cell lines was completely eliminated after sunitinib treatment and the level of Tregs was restored to baseline (0.5 ± 0.2%; p < 0.01 and 0.4 ± 0.1%; p < 0.01, respectively).

These data demonstrate that sarcoma cells loaded in DCs have a tolerogenic effect on T cells thus leading to strong Treg induction, while sunitinib contrasts this sarcoma tolerogenic feature, restoring the values to those observed with unstimulated T cells.

### PD-1 Blockade by Nivolumab Synergizes With Sunitinib in Inducing IFN-γ-Producing Effector T Cells

Moving on from the observation that sunitinib is capable of upregulating PD-L1 expression on treated sarcoma cells (**Figure 1D**) and given its described immunomodulatory properties, we sought to test the hypothesis that PD-1 blockade by nivolumab might be synergistic with sunitinib in the stimulation of T cells. To demonstrate this hypothesis, the SaOS-2 and SYO-1 cell lines were first treated with sunitinib (20 μM and 15 μM, respectively) and pulsed into DCs, which were used to stimulate CD3^+^ T cells for 5 days. The PD-1 expression on total CD4^+^ and CD8^+^ T cells, T-cell subpopulations such as naïve (N), central memory (CM), effector memory (EM) and EM expressing RA (EMRA) and Tregs was then evaluated.

**Figure 5:**
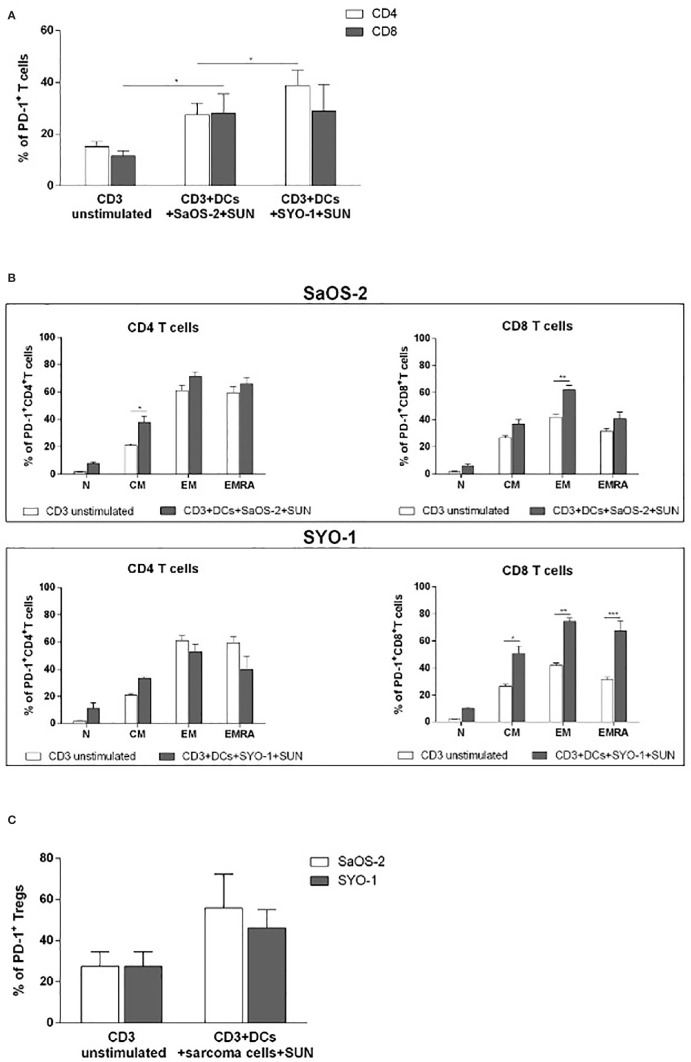
Flow cytometry analysis of PD-1 expression on T cells induced by DCs loaded with sunitinib treated sarcoma cells. SaOS-2 and SYO-1 cell lines were treated for 24 h with sunitinib (SUN) at concentrations of 20 μM and 15 μM, respectively, and used for monocyte-derived DC pulsing for another 24 h at a ratio of 1:2 (DC:sarcoma cell). Then, DCs loaded with untreated and irradiated SaOS-2/SYO-1, or sunitinib-treated cells (DCs+SaOS-2/SYO-1+SUN) were mixed with autologous CD3^+^ T cells at a ratio of 1:10 (DC:T cell) After 5 days, the percentage of PD-1 on **(A)** CD4^+^ and CD8^+^ T cells, **(B)** naïve (N), central memory (CM), effector memory (EM), and EM expressing RA (EMRA) CD4^+^ and CD8^+^T cells and **(C)** Tregs was evaluated by flow cytometry. The values are represented as mean ± SEM of 5 independent experiments. *p < 0.05; **p < 0.01; ***p < 0.001.

As shown in **Figure 5A**, DCs loaded with sunitinib-treated SaOS-2 and SYO-1 cell lines induced an upregulation of PD-1 expression on both CD4^+^ and CD8^+^ T cells as compared to unstimulated T cells (p < 0.05 for both cell lines).

**Figure 6:**
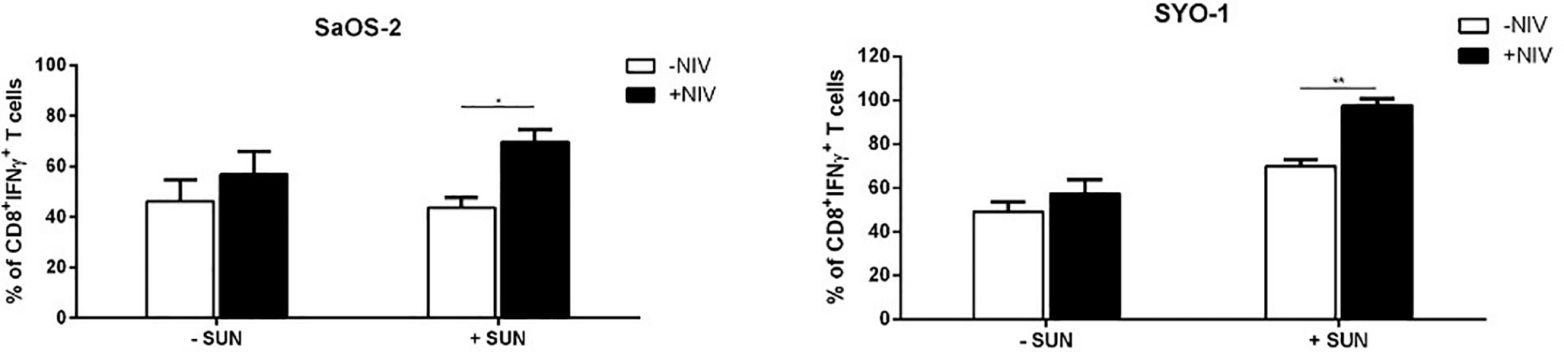
Flow cytometry analysis of nivolumab effect on PD-1 expression in sarcoma-specific CD8^+^ T cells activated by PMA/IM. SaOS-2 and SYO-1 cell lines were left untreated or treated with sunitinib for 24 h at concentrations of 20 μM and 15 μM, respectively, and used for monocyte-derived DC pulsing for another 24 h at a ratio of 1:2 (DC:sarcoma cell). DCs loaded with untreated SaOS-2/SYO-1 (- SUN) or sunitinib-treated SaOS-2/SYO-1 cells (+ SUN) were then used to stimulate and restimulate autologous CD3^+^ T cells at a ratio of 1:10 (DC:T cell). 24 h after restimulation, the stimulated CD3^+^ effector T cells were activated by PMA/IM in the presence or absence of nivolumab (20 µg/ml) for 4 h and the reaction was stopped by adding Brefeldin A. The percentages of IFN-γ-producing CD3/CD8^+^ T cells were evaluated by flow cytometry. The values are represented as mean ± SEM of 3 independent experiments; *p < 0.05; **p < 0.01.

Next, we characterized the differential expression of PD-1 expression on T-cell subpopulations, including Tregs, after cultures with DCs, previously pulsed with sunitinib-treated cell lines. As shown in **Figure 5B**, a significant PD-1 upregulation was induced by sunitinib-treated SaOS-2 cell line-loaded DCs in CD4^+^ CM and CD8^+^ EM T cells (p < 0.05 and p < 0.01, respectively), when compared to unstimulated CD4^+^ and CD8^+^ T cells, respectively. As for the SYO-1 cell line, a significant upregulation of PD-1 expression was observed in CM, EM and EMRA CD8^+^ T cells induced by DCs loaded with sunitinib-treated SYO-1 cells when compared to unstimulated CD8^+^ T cells (p < 0.05, p < 0.01 and p < 0.001, respectively). Regarding Tregs, as shown in **Figure 5C**, sunitinib-treated SaOS-2 and SYO-1 cell line-loaded DCs, when used for CD3^+^ T cell stimulation, induced only a trend of PD-1 upregulation on Tregs compared to unstimulated T cells. To demonstrate a synergistic effect of PD-1 blockade with sunitinib, we evaluated the effect of nivolumab on IFN-γ production by effector T cells *via* DCs loaded with sunitinib-treated sarcoma cells. Firstly, the sunitinib-treated SaOS-2 (20 μM) and SYO-1 (15 μM) cells were loaded into DCs, which were in turn used to stimulate autologous CD3^+^ T cells for inducing the IFN-γ production by CD8^+^T cells. After cultures, T cells were activated by PMA/IM in the presence or absence of nivolumab (20 μg/ml), and IFN-γ-producing CD8^+^ T cells were analyzed by flow cytometry. As shown in **Figure 6**, a statistically significant increase of IFN-γ-producing CD8^+^ T cells was observed when CD3^+^ T cells stimulated with DCs loaded with sunitinib-treated SaOS-2 and SYO-1 cells were cultured in the presence of nivolumab as compared to the condition without nivolumab (p < 0.05 and p < 0.01, respectively). The effect of nivolumab was observed when T cells were cultured with DCs loaded with sunitinib-treated tumor cells, suggesting an important role of sunitinib treatment in this process. Indeed, no increase in IFN-γ-producing CD8^+^ T cells was observed for T cells stimulated with DCs loaded with untreated SaOS-2 cells and SYO-1 cells (p > 0.05).

Taken together, these results indicate a synergistic immunological activity of sunitinib and nivolumab. In particular, sunitinib induces a DC-based cross-priming effect of IFN-γ-producing effector T cells and this effect is increased by blocking PD-1 with nivolumab.

## Discussion

In the last few years, an increasing body of evidence has highlighted the immunomodulatory effect of some antineoplastic agents, i.e., chemotherapeutics and radiotherapy which, along with inducing an antiproliferative effect on tumor cells, may concomitantly act as adjuvants of the immune system. In this process, known as immunogenic cell death (ICD), tumor-infiltrating DCs play a major role by capturing tumor antigens from chemotherapy-treated dying tumor cells and then orchestrating the fine-tuned activation and expansion of tumor-specific effector T cells (20). An impairment of this effect mediated by DCs has been correlated with a reduced efficacy of chemotherapy in a wide variety of tumor types (20) and with a worse clinical outcome of cancer patients (20, 21). These data indicate that a more-in-depth characterization of the DC-driven immunomodulatory effect of antineoplastic treatments has important clinical implications and should be taken into consideration in the design of new combination treatments, which include immunotherapy agents. It is known that sunitinib may have important immunological effects (13–15), but no data are available about its capacity to modulate the phenotype and function of DCs and then favor the cross-priming effect on T cells in sarcomas. Our *in vitro* results expand our knowledge on the immunomodulatory profile of sunitinib on sarcoma cells by providing evidence of its effect as an immune adjuvant through DCs. Sunitinib-treated sarcoma cells drive DCs to full maturation and increase their capacity to induce sarcoma-reactive T cells to produce IFN-γ. To formally demonstrate that ICD-related events in sarcoma cells, such as the induction of inflammatory pathways, might be involved in the sunitinib effect on DCs was beyond the scope of this study. However, one recent paper reported the capacity of TKIs, such as crizotinib, to induce ICD in various tumor cells, including sarcoma cell line U2OS (22). These findings suggest that other TKIs, i.e., sunitinib, may act as immune adjuvants by prompting dying sarcoma cells to acquire immunogenic properties.

Interestingly, when loaded with sunitinib-treated sarcoma cells, DCs also have a reduced capacity to induce Tregs. During ICD, Treg induction is known as an undesirable bystander effect, which may significantly hamper effector T-cell activation. We recently demonstrated that, while activating cytotoxic T lymphocytes, chemotherapy-treated acute myeloid leukemia cells may also potently induce immune tolerance through the expansion/induction of tolerogenic DCs and Tregs (17, 23). In this scenario, our finding that in sarcoma cells sunitinib treatment may reduce the tolerogenic propensity of DCs to induce Tregs is in line with previous reports on the capacity of sunitinib to increase the influx of lymphocytes and DCs into the tumor, while decreasing the intratumoral frequencies of Tregs and MDSCs (13–15). Of note, this is the first demonstration of such an effect in sarcoma cells and, along with the results indicating the capacity of sunitinib to increase DC-based sarcoma-reactive T cells, may provide further rationale for choosing sunitinib as an ideal drug to be combined with immunotherapies in the treatment of sarcomas. *In vitro* studies may have important limitations. The most relevant of these are the discrepancy between drug concentrations that are clinically relevant and those used in *in vitro* modeling as well as the off-target effects of the drug on other cells, i.e., immune cells. Specifically, in the clinical setting the serum concentrations of sunitinib are lower than those used in our *in vitro* model. However, our *in vitro* model of cross-priming through DCs relies on the capacity of sunitinib to induce a certain degree of apoptosis in treated tumor cells, which is obtained only by using higher concentrations. A similar approach was used by our group in a previous study (17) and by other groups (22). Although such concentrations may affect *in vivo* the viability of immune cells, in our experiments of cross-priming *via* DCs, the immune cell subsets are not directly exposed to sunitinib and as previously discussed, the concentrations of sunitinib used in the clinical setting are lower and safer for immune cells.

Several pre-clinical studies and clinical trials have been performed on the combination of anti-angiogenic compounds, sunitinib among them, and anti-immune checkpoint drugs in sarcoma (24), and other solid tumors (25, 26). The clinical efficacy and tolerability of TKIs and checkpoint inhibitors in soft-tissue sarcomas was demonstrated (8, 27), with a better overall response rate and progression-free survival in alveolar sarcomas. Only recently, have the results of a nivolumab and sunitinib combination in patients with bone sarcoma been presented, with an objective response in dedifferentiated chondrosarcomas and bone sarcomas (12). These findings are in line with recently published results of clinical trials in kidney cancer, demonstrating superior progression-free survival and overall survival for an anti-VEGFR and anti-PD-L1 combinatorial approach both over sunitinib (28–30) and checkpoint inhibitor monotherapy (31). Our *in vitro* results confirm the potential of combining sunitinib with PD-1 blockade and expand our knowledge on the potential mechanisms underlying the combination of sunitinib and immune checkpoint inhibitors, suggesting a central role for DCs in regulating the expression of immune checkpoint receptors on T-cell subsets after sunitinib treatment. Indeed, after treatment with sunitinib, sarcoma cell lines upregulate PD-L1 expression which is in line with a similar observation in recent RCC studies. Moreover, for the first time, it was shown that sunitinib-treated sarcoma cells drive DCs to upregulate PD-1 expression on a wide variety of T cell subsets. Importantly, CD8^+^ T cells are induced to produce IFN-γ by DCs previously pulsed with sunitinib-treated sarcoma cells and this effect is significantly increased by PD-1 blocking with nivolumab. With the limitation of an *in vitro* study, these data support the hypothesis that sunitinib and nivolumab may have a synergistic immunological potential through the modulation of DC-based cross-priming of effector T cells. To corroborate these findings, studies in the mouse model are warranted. In mice, we and others reported the capacity of chemotherapy to impact on the composition of the tumor infiltrate by modulating the phenotype and function of infiltrating DCs (17, 20, 21). These effects lead to the expansion of a population of tumor-specific T cells, which have a crucial role in preventing the growth of tumor cells at subsequent tumor challenge. Starting from the well-established capacity of sunitinib to profoundly subvert the composition of the tumor infiltrate (15, 16, 32), a similar set of experiments in sarcomas will make it possible to *in vivo* envisage the contribution of DCs in such a process, highlighting the potential of sunitinib as an immune adjuvant.

DC-targeted drugs, including toll-like receptor 3 and CD47 inhibitors, represent a novel class of compounds focused on the adaptive immune system that might succeed in circumventing immune evasion (33). Recent advances in synthetic biology and the increasing understanding of the cluster of differentiation 47/signal regulatory protein alpha (CD47/SIRP7) axis may provide new opportunities for the clinical application of engineered macrophages. Many pediatric tumors, including bone sarcomas and synovial sarcomas, demonstrate surface expression of SIRPα on a par with that of CD47, suggesting that the interaction between the two is likely relevant. Clinical trials with anti-CD47 in patients with sarcomas are ongoing (34). Our results might contribute to the understanding of immunomodulation *via* DCs.

## Conclusions

Immunomodulation represents one of the major achievements in cancer treatment, with the approval of several drugs in the last decade. Unsatisfactory results in the so called *immune cold* tumors, including sarcomas, suggest that combinatorial approaches are needed. Our results confirm that sunitinib is able to improve nivolumab activity through DC maturation and might induce tolerance through Tregs in a preclinical model of sarcoma. DC-targeted drugs, including toll-like receptor 3 and CD47 inhibitors, are under development and our preclinical model might help to better design their clinical application.

## Data Availability Statement

The original contributions presented in the study are included in the article/supplementary material. Further inquiries can be directed to the corresponding author.

## Ethics Statement

The studies involving human participants were reviewed and approved by Comitato Etico di Area Vasta Emilia Centro della Regione Emilia-Romagna (CE-AVEC). The participants provided their written informed consent to participate in the study.

## Author Contributions

Conceptualization, AC, DO and EP; methodology, DO and ML; software, DO; validation, DO, EP and AC; formal analysis, DO; resources DO and AC; data curation, DO; writing—original draft preparation, DO, AC and EP; writing—review and editing, DO, AC, JMB and MC; supervision, AC, EP and KS; funding acquisition, AC. All authors contributed to the article and approved the submitted version.

## Funding

This study was supported by the Associazione Onlus ‘il Pensatore: Matteo Amitrano’, by Bologna AIL (Associazione Italiana contro le Leucemie), Section of Bologna; FATRO, Foundation Corrado and Bruno Maria Zaini-Bologna.

## Conflict of Interest

EP has served on advisory boards for Amgen, Daiichi Sankyo, Lilly, Deciphera, Eusa Pharma, received other research support from Bristol Myers Squibb, Pfizer, PharmaMar, Daiichi Sankyo, Incyte, and travel support from Lilly, PharmaMar, and Takeda. AC received honoraria from Novartis, Pfizer, Abbvie and acted as speaker on the Advisory Board for Novartis and Abbvie. JB reports research expenses from GlaxoSmithKline-Novartis, grants and personal fees from Novartis and Pharmamar, grants from EISAI, and personal fees from Lilly, outside the submitted study.

The remaining authors declare that the research was conducted in the absence of any commercial or financial relationships that could be construed as a potential conflict of interest.
